# Image-and-text health warning labels on alcohol and food: potential effectiveness and acceptability

**DOI:** 10.1186/s12889-020-8403-8

**Published:** 2020-04-02

**Authors:** Emily Pechey, Natasha Clarke, Eleni Mantzari, Anna K. M. Blackwell, Katie De-Loyde, Richard W. Morris, Theresa M. Marteau, Gareth J. Hollands

**Affiliations:** 1 Behaviour and Health Research Unit, Institute of Public Health, University of Cambridge, Forvie Site, Cambridge, CB2 0SR UK; 2 Tobacco and Alcohol Research Group, University of Bristol, School of Psychological Science, Bristol, BS8 1TU UK; 3 Bristol Medical School: Population Health Sciences, Canynge Hall, 39 Whatley Road, Bristol, BS8 2PS UK

**Keywords:** Graphic health warning labels, Pictorial health warning labels, Image-and-text warning labels, Cancer, Alcohol, Food

## Abstract

**Background:**

Health warning labels (HWLs) using images and text to depict the negative health consequences of tobacco consumption are effective and acceptable for changing smoking-related outcomes. There is currently limited evidence concerning their potential use for reducing consumption of alcoholic drinks and energy-dense foods. The aim of this research was to describe the potential effectiveness and acceptability of image-and-text (also known as pictorial or graphic) HWLs applied to*: i.* alcoholic drinks and *ii.* energy-dense snack foods.

**Methods:**

Two online studies were conducted using between-subjects designs with general population samples. Participants rated one of 21 image-and-text HWLs on alcoholic drinks (*n* = 5528), or one of 18 image-and-text HWLs on energy-dense snacks (*n* = 4618). HWLs comprised a graphic image with explanatory text, depicting, respectively, seven diseases linked to excess alcohol consumption, and six diseases linked to excess energy intake. Diseases included heart disease and various cancers. Outcomes were negative emotional arousal, desire to consume the labelled product, and acceptability of the label. Free-text comments relating to HWLs were content analysed.

**Results:**

For both alcoholic drinks and energy-dense snacks, HWLs depicting bowel cancer generated the highest levels of negative emotional arousal and lowest desire to consume the product, but were the least acceptable. Acceptability was generally low for HWLs applied to alcohol, with 3 of 21 rated as acceptable, and was generally high for snacks, with 13 of 18 rated as acceptable. The majority of free-text comments expressed negative reactions to HWLs on alcohol or energy-dense snacks.

**Conclusions:**

Image-and-text health warning labels depicting bowel cancer showed greatest potential for reducing selection and consumption of alcoholic drinks and energy-dense snacks, although they were the least acceptable. Laboratory and field studies are needed to assess their impact on selection and consumption.

## Introduction

Alongside tobacco, excess consumption of alcohol and of energy-dense foods are two of the most significant preventable causes of a range of non-communicable diseases globally, including heart disease and many cancers [1–3]. Both products significantly contribute to energy intake, with alcohol being energy-dense and on average accounting for an estimated 8.8% of total energy intake in drinkers [4]. Based on evidence from the field of tobacco cessation, health warning labels (HWLs) have the potential to reduce harmful consumption.

HWLs that highlight the negative health consequences associated with consumption are currently mandated for use on tobacco packaging in 118 countries worldwide [5], including the UK, covering over half of the world’s population. There is a substantial body of evidence showing their effectiveness on a range of outcomes including cessation-related behaviours [6]. Evidence indicates image-and-text (also known as ‘pictorial’ or ‘graphic’) HWLs - i.e. those that contain an image alongside text - are more effective than text-only HWLs [7–9] across socio-economic groups [10]. Given clear evidence that HWLs on tobacco are a feasible and effective population-level intervention, there is considerable interest in applying them to other potentially health-damaging products, in particular, alcoholic and sugar-sweetened beverages (SSBs), and foods high in saturated fat, sugar and salt [11, 12]. Uncertainty remains, however, around the design of HWLs with the potential to reduce consumption of these products, as well as the public acceptance of such labels.

Evidence regarding the impact of HWLs on alcohol and food products is limited [13]. The very few studies investigating image-and-text HWLs on alcohol products are promising, with such HWLs slowing consumption [14] and reducing intentions to drink [15]. However, these studies are based on very small sample sizes. For food, text-only HWLs have been shown to decrease intentions to consume and purchase a range of labelled food products [16], and decrease the likelihood of purchasing SSBs [17]. Image-and-text HWLs on food have also been shown to increase dietary self-control in relation to snack foods [18, 19], reduce hypothetical selection [17, 20], and real-life purchasing of SSBs [21]. Comparing different types of HWLs, image-and-text HWLs are more effective at reducing selection of SSBs than text-only HWLs, nutritional labels [17, 21], or labels depicting sugar content [20].

Research from tobacco suggests that image-and-text HWLs can increase quit attempts, through eliciting negative emotions – such as fear, disgust, discomfort and worry [22]. Similarly, preliminary research shows that image-and-text HWLs increase fear arousal and intentions to reduce alcohol consumption compared to text-only HWLs [15]. In addition, disgust has been identified as a key component of the effects of alcohol HWLs on intentions to reduce alcohol intake [23]. In the context of food, negative emotional arousal has been highlighted as a potential mediator of the effects of HWLs on SSB selection [20]. However, the two studies to date that have investigated the effect of food HWLs on negative emotions specifically concern SSBs [20] or were conducted in unrepresentative populations [24].

In addition to its effectiveness, the public acceptability of an intervention affects the likelihood that it is implemented [25]. Public attitudes can also change with evidence of an intervention’s effectiveness [21, 26, 27]. Public support for tobacco control policies, such as taxation and image-and-text HWLs, is generally high [26, 28]. This high acceptability reflects the low prevalence of smoking in the population, with people generally less supportive of policies that might affect their own behaviour [26]. It may also reflect a high awareness of tobacco harms [28] and exposure to image-and-text HWLs [10]. Research into the acceptability of HWLs in the context of alcohol and food is limited [25]. A recent study found high acceptability for the implementation of image-and-text HWLs for both alcohol and food [26], but respondents were not shown examples of the warnings. A small number of studies suggest that text-only HWLs are generally accepted for both alcohol [29] and SSBs [30] but image-and-text HWLs could plausibly be less acceptable due to their graphic nature. While studies have investigated the acceptability of drinks displaying image-and-text HWLs [14] to our knowledge no studies have investigated the acceptability of the labels themselves in the context of alcohol. In the context of SSBs, in the UK, image-and-text HWLs were found to be less acceptable than labels depicting sugar content or calorie information [20]. In New Zealand, they were less acceptable than text-only HWLs – with 66% support for the introduction of text-only HWLs on SSBs, compared with 50% support for image-and-text HWLs [17]. However, both of these studies were based on HWLs on SSBs, which may differ from the acceptability of HWLs on food and alcohol, due to an increased awareness of the harms of SSBs, with legislative bills introduced in several US jurisdictions requiring the introduction of text-only warnings on SSBs (e.g. New York State Assembly Bill 2320-B). As such, varying results observed across studies may reflect differences both in product contexts, the content or form of HWL, as well as the different means of assessing acceptability.

Overall, the evidence concerning behavioural and affective responses to image-and-text HWLs on alcoholic drinks and snack foods is limited in both quantity and scope, meriting further investigation. The aim of the current two studies is to assess the potential effectiveness and acceptability of image-and-text HWLs applied to: *i*. alcoholic drinks and *ii.* energy-dense snack foods.

## Methods

Two online studies were conducted with separate sets of participants, one focusing on alcoholic drinks (Alcohol study) and one on energy-dense snack foods (Food study). These are described in turn.

### Alcohol study

#### Preregistration

The study protocol was preregistered on the Open Science Framework, prior to data collection: https://osf.io/pr8zu/.

#### Design

A between-subject design was used, with participants randomised to view one of 21 image-and-text HWLs on an alcoholic drink.

#### Setting

The study was conducted online using the Qualtrics survey platform.

#### Participants

Participants (*n*= 5528) were recruited through a market research agency (Dynata) and  purposefully sampled from the general UK population to include a range of age, gender, and social grades. Eligible participants were those aged 18 or more, fluent in English, with access to a computer, who self-reported consuming either beer or wine at least once a week. Potential participants were registered with the research agency. All registered participants over 18 years old were invited to take the survey via email or could access the survey platform directly through the research agency website. Eligibility to participate was then determined via screening questions at the beginning of the survey. Only participants who consumed the target products were included, to reflect the likely principal target population for any comparable intervention applied in a real world setting.

### Sample size determination

The current study was powered conservatively to detect a small difference between any pair of the conditions (Cohen’s *d* = 0.25) with power = 0.8, and alpha = 0.05, requiring 256 participants for each of 21 conditions, giving a total minimum sample size of 5376 participants.

#### Materials

Each image-and-text HWL comprised an image depicting a health outcome, i.e. various cancers or heart disease, accompanied by text describing that outcome. A single graphic image was used for each HWL, defined as a photographic representation of the human body’s structure, anatomy or pathology (such as damaged organs or scenes of surgery). The full range of alcohol HWLs are available online: https://osf.io/pr8zu/.

Images used for the HWLs were chosen from a pool of 47 possible images sourced from previous studies [31] and online image databases such as Science Photo Library and Shutterstock. These comprised images of seven health consequences linked to alcohol consumption including bowel cancer, breast cancer, liver cancer, cancer (non-specified), heart disease, liver cirrhosis and liver disease. A convenience sample of 15 colleagues, working in the fields of public health, psychology and statistics, ranked the 47 different images based on the image’s perceived impact in deterring individuals from consuming excess alcohol. The three images ranked most highly for each of the seven health consequences were selected. Accompanying text was developed based on text used in previous studies and evidence about designing effective messages [2, 15, 19, 20, 32, 33]. See Table 1.

**Table 1:** Health consequences depicted in HWLs in the alcohol and food studies for the different health consequences used for the Alcohol and Food studies.

Health consequences^**a**^	Alcohol Study “Alcohol causes …”	Food Study “Excess calories cause obesity, which causes) …”
Non-specified cancer		X
Bowel cancer	X	X
Breast cancer	X	
Liver cancer	X	
7 types of cancer	X	
13 types of cancer		X
Heart disease	X	X
Liver disease	X	
Liver cirrhosis	X	
Type 2 diabetes		X
Obesity^b^		X

^a^Three HWLs with different images were used for each health consequence.

^b^The text read ‘Excess calories cause obesity’ without the additional clause.

### Measures

#### Primary outcome measure

##### Negative emotional arousal

This was assessed using a four-item measure (Cronbach’s α = 0.89) previously used to assess the impact of HWLs on cigarette packages [34] and on sugar-sweetened beverages [20]. Responses to each of the four items are rated on a seven-point scale: How [afraid/worried/ uncomfortable/disgusted] does the label on this drink make you feel?’ (0 Not at all [afraid/worried/uncomfortable/disgusted] to 7 very [afraid/worried/uncomfortable/ disgusted]).

#### Secondary outcome measures

##### Desire to consume the labelled product

This was assessed using a single-item seven-point measure: ‘How much do you want to drink this (wine or beer) right now?’ (0 Not at all to 7 very much).

##### Acceptability of health warning labels

This was assessed using a single-item seven-point measure adapted from previous studies [27] ‘Do you support or oppose putting this label on alcoholic drinks?’ (Strongly oppose – neither oppose nor support – strongly support). Ratings past the midpoint, i.e. above 4 on the scale, were taken to indicate that the label was acceptable.

##### Free-text responses

A free-text box was provided at the end of the study into which participants were invited to write comments (‘Do you have any further thoughts or comments that you would like to add?’). See Analysis section for details.

#### Additional measures

Age, gender, ethnicity, education level, household income, height and weight were all self-reported. Participants also reported their typical alcohol consumption. These measures were collected for the purpose of describing the sample only.

#### Procedure

Ethical approval was granted by the Cambridge Psychology Research Ethics Committee (PRE.2018.071). Participants were informed at the start of the study that they could withdraw at any point. After providing written consent to take part via an online consent form at the beginning of the study, participants completed screening questions relating to their typical consumption of alcohol and their age. Participants who reported drinking less than once a week on average, or were under 18 years old, were screened out (see ‘Participants’). Those screened out were diverted to a webpage that explained that they were ineligible for taking part in this study, and then redirected to the research agency home page. Eligible participants were then asked questions regarding their demographic characteristics (gender, ethnicity, education level, household income, height and weight). In order to ensure that images were clearly visible, the study was conducted on computer-size screens. Participants accessing the study on mobile phones were identified by the Qualtrics software and automatically screened out as soon as they clicked on the study link. Inattentive participants were screened out via an embedded attention check (asking participants, ‘when was the last time you travelled to Mars?’ [months ago/weeks ago/a few days ago/ never] [20, 27], with any participant who responded anything other than ‘never’ being screened out).

**Fig. 1:**
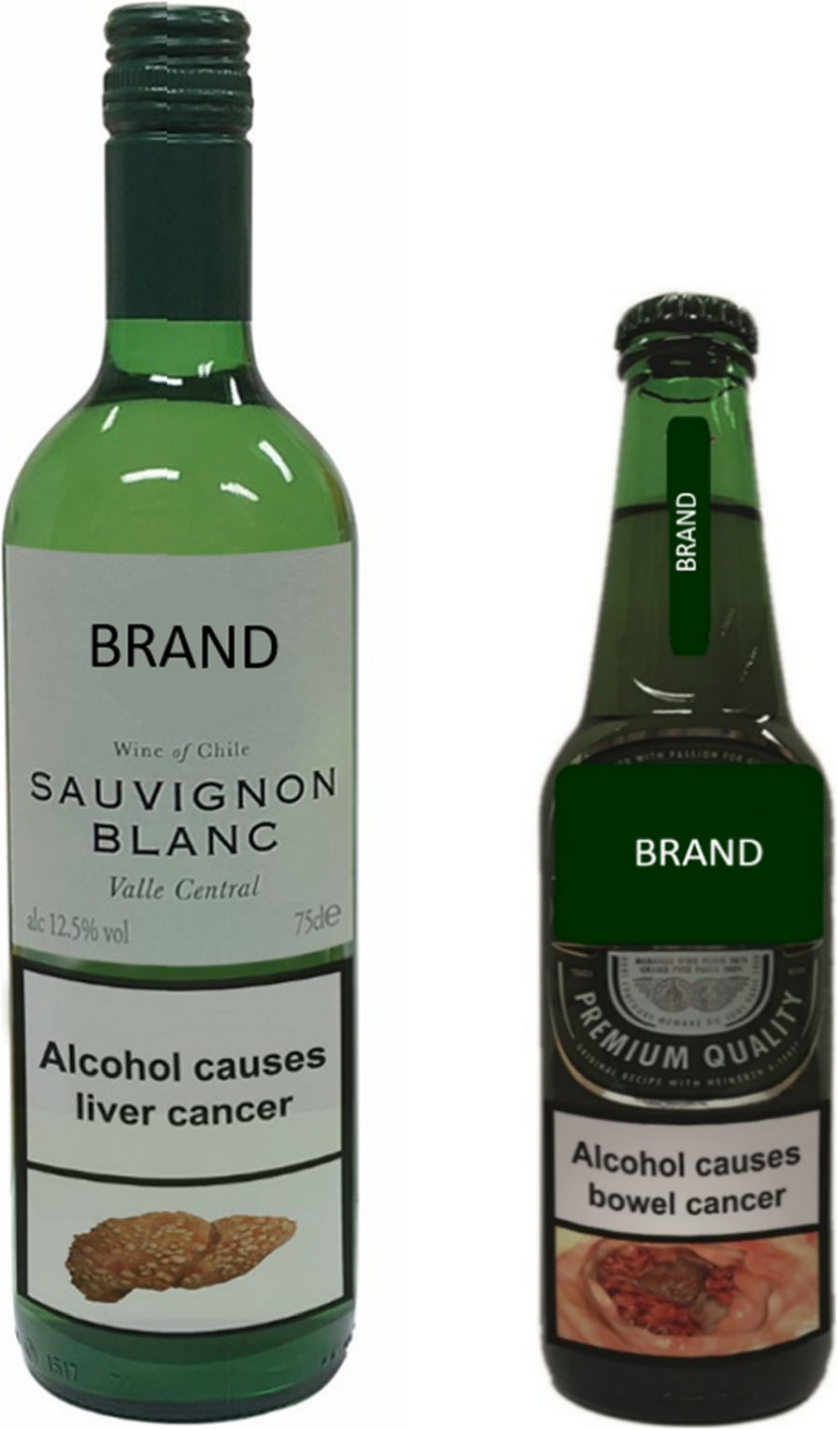
Example labelled products. The HWL was presented on beer or wine depending on participants’ stated preference. The drinks used in the study were branded, but product branding is covered here for copyright reasons. A single brand of wine or beer was used consistently across participants – to ensure effects would be due to different HWLs and not due to different brands. Image permissions from Shutterstock (https://www.shutterstock.com)

Participants viewed an image of beer or wine (depending on their stated preference) displaying, at random, one of 21 different image-and-text HWLs illustrating the adverse health consequences of alcohol consumption (see Fig. 1 for example). Randomisation to the different HWLs was completed by an algorithm within the Qualtrics online platform. After viewing the HWL, participants completed the measures of negative emotional arousal, desire to consume the labelled product and acceptability. Finally, participants had the opportunity to leave a comment in a free-text box. Upon completion, participants were debriefed, which included providing information about the health consequences of consuming excess alcohol, and were reimbursed for their participation. Data were collected from October to December 2018.

### Analysis

#### Descriptive analysis

Following normality checks, the mean and standard deviation (SD) and 95% confidence intervals (CIs) were calculated for each outcome and each HWL. An analysis plan was pre-registered before the data were inspected (https://osf.io/pr8zu/).

#### Content analysis

Comments provided by participants were manually coded and two emergent themes were identified. Responses were coded into themes, and as being either positive, neutral or negative. For full details of the analytic procedure see Additional file 1.

### Food study

#### Preregistration

The study protocol was preregistered on the Open Science Framework, prior to data collection: https://osf.io/k7tw5/.

#### Design

Participants were randomised to view one of 18 image-and-text HWLs on an energy-dense snack.

#### Setting

The study was conducted online using the Qualtrics survey platform.

#### Participants

Recruitment process, sample and eligibility criteria were identical to that of the Alcohol Study (see above). The sole difference was the target product consumed - participants were self-reported regular consumers of biscuits, cake, crisps or chocolate (i.e. consumed at least once a week), and liked chocolate.

##### Sample size

The same sample size information was used as for the Alcohol study (with 256 participants needed per condition). With 18 conditions, a sample size of at least 4608 participants was required.

#### Materials

HWL images were selected from a pool of 33 images depicting six different health consequences, including bowel cancer, cancer (non-specified), heart disease, obesity and type 2 diabetes. The form comprised a graphic image plus text statement. For each of the six health consequences (see Table 1), three image-and-text HWLs were developed. The process of developing the HWLs and piloting them was the same as that described for the Alcohol study. The full range of food HWLs are available online: https://osf.io/k7tw5/.

#### Measures

The measures used were identical to those used in the Alcohol Study, with the only differences being on three measures that named the product on which the label was placed, which was changed from alcohol to snack:

#### Primary outcome measure

##### Negative emotional arousal

Four-item measure ‘How [afraid/worried/uncomfortable/disgusted] does the label on this snack make you feel?’ (Cronbach’s α = 0.91)

#### Secondary outcome measures

##### Desire to consume the product

‘How much do you want to eat this snack right now?’

##### Acceptability

‘Do you support or oppose putting this label on high calorie snacks?’

In an identical method as in the Alcohol study, free-text responses were also collected at the end of the study.

#### Additional measures

Age, gender, ethnicity, education level, household income, height and weight were all self-reported. Participants also reported their typical consumption of energy-dense snacks. These measures were collected for the purpose of describing the sample only.

#### Procedure

Ethical approval was granted by the Cambridge Psychology Research Ethics Committee (PRE.2018.072). As in the Alcohol study, participants were informed at the start of the study that they could withdraw at any point and gave written consent. Participants used computer-size screens for enhanced visibility of the HWLs. Participants completing the study on mobile phones were screened out. After consenting, participants completed screening questions relating to age, their typical consumption of energy-dense snacks, and whether they liked chocolate. If they did not eat an energy-dense snack at least once a week, did not like chocolate, or were under 18 years old, they were screened out. Participants then answered demographic questions, with an embedded attention check (identical to Alcohol study procedure - see above).

**Fig. 2:**
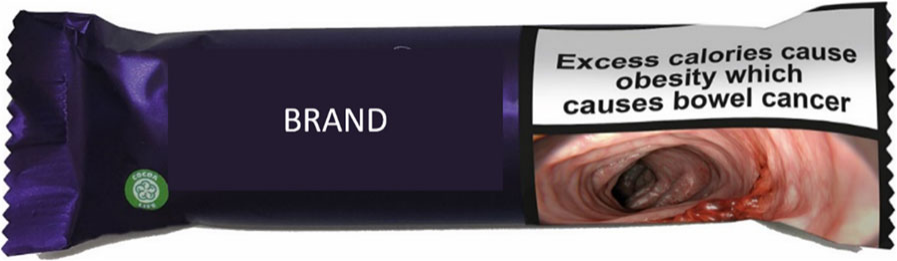
Example labelled product. The snack used in the study was a popular branded chocolate bar, but product branding is covered here for copyright reasons. A single brand was used consistently across participants – to ensure effects would be due to different HWLs and not due to different brands. Image permissions from Shutterstock (https://www.shutterstock.com)

Participants viewed an image of a chocolate bar illustrating 18 different image-and-text HWLs depicting the adverse health consequences of obesity and related conditions, caused by excess calorie consumption (see Fig. 2 for example) and rated them on negative emotional arousal, desire to consume the snack and acceptability of the HWL. Randomisation to the different HWLs was completed by an algorithm within the Qualtrics online platform. Finally, participants had the opportunity to leave a comment in a free text box. Upon completion, participants were debriefed, which included providing information about the health consequences of consuming excess calories, and reimbursed. Data were collected from October to December 2018.

#### Descriptive and content analysis

Both descriptive and content analysis procedures were identical to the Alcohol study (see above). The analysis plan was preregistered: https://osf.io/k7tw5/.

## Results

### Participants

#### Alcohol study

In total 5953 eligible participants took the online survey and 425 participants dropped out, leaving a final sample size of *n* = 5528. These final numbers were higher than the necessary minimum sample size, as we over-recruited to account for potential dropout. Each HWL was rated by a minimum of 259 participants. On average, participation took 8 min, (mean = 8 min, 27 s). Approximately half of the sample (50.9%) were female and had a mean age of 47.5 (SD = 15.8). The sample included participants from a range of income and education levels (44.8% were educated to degree level or above) and BMI (43.2% in the healthy weight category). See Table 2 for participant demographics*.*

**Table 2:** Participants’ demographic characteristics (% (n), unless stated otherwise)

Measure	Alcohol Study (*n* = 5528)	Food Study (*n* = 4618)
Age (years)		
Mean (SD)	47.5 (15.8)	47.5 (16.1)
BMI (kg/m^2^)		
Mean (SD)	26.3 (5.6)	26.8 (6.1)
Underweight (under 18.5)	3.5 (190)	3.8 (172)
Healthy weight (18.5–24)	43.2 (2337)	39.6 (1782)
Overweight (25–29)	32.1 (1737)	31.7 (1425)
Obese (30–34)	13.7 (744)	14.6 (657)
Severely obese (35–39)	4.7 (257)	6.1 (283)
Morbidly obese (40 or more)^a^	2.7 (148)	4.0 (182)
Gender		
Male	49.1 (2709)	49.1 (2267)
Female	50.9 (2807)	50.7 (2340)
Other	0.1 (4)	0.2 (8)
Ethnicity		
White	93.9 (5193)	90.4 (4150)
Mixed/Multiple Ethnic Groups	1.5 (82)	2.2 (99)
Asian/Asian British	2.7 (147)	4.9 (223)
Black/African/Caribbean/Black British	1.2 (68)	2.1 (98)
Other ethnic group	0.3 (14)	0.4 (19)
Highest qualification		
None	4.2 (233)	5.7 (262)
Up to 4 GCSEs	13.0 (717)	14.3 (656)
5 or more GCSEs or 1 A-level (Including 5+ GCSEs	15.9 (878)	14.9 (683)
2 or more A-levels	18.7 (1030)	19.2 (880)
Bachelor’s degree	29.4 (1618)	28.1 (1293)
Post-Graduate degree or qualification	15.4 (846)	14.0 (645)
Other vocational/work-related qualifications	3.3 (184)	3.8 (175)
Income (per year)		
Up to £11,499	8.4 (541)	14.0 (645)
£11,500 – £24,999	21.3 (1138)	24.5 (1130)
£25,000 - £49,999	41.8 (2237)	35.3 (1628)
£50,000 or more	23.1 (1230)	18.2 (841)

^a^BMI categories based on WHO guidelines [35]

#### Food study

In total 4905 eligible participants took the online survey and 287 participants dropped out, leaving a final sample of *n* = 4618. These final numbers were higher than the necessary minimum sample size, as the sample was over-recruited to account for potential dropout. Each HWL was rated by a minimum of 255 participants. On average, participation took 6 min (mean = 6 min, 9 s). Approximately half of the sample were female (50.7%) and had a mean age of 47.5 (SD = 16.1). The sample included participants from a range of income and education levels (42.1% were educated to degree level or above) and BMI (39.6% in the healthy weight category). See Table 2 for participant demographics.

### Descriptive analysis

#### Alcohol study (see Table 3 and Fig. 3)

The health consequence that elicited the highest negative emotional arousal (NEA) was bowel cancer, followed by liver cancer. Labels depicting bowel cancer elicited higher levels of NEA than those depicting all other health consequences, with no overlap in the respective 95% confidence intervals (CIs). HWLs relating to bowel cancer were also on average rated lowest in desire to consume the product, although all 95% CIs overlapped. In general, few of the alcohol HWLs were considered acceptable, with only 3 out of 21 individual HWLs rated as at least somewhat acceptable (with a mean rating above 4 out of 7). Bowel cancer related HWLs were overall rated least acceptable, with 95% CIs not overlapping with any labels other than those depicting breast cancer and heart disease.

**Table 3:** Alcohol: Means (±SDs) for NEA, desire and acceptability by label and health consequence [ranked from highest to lowest, 1–21]

Label	Negative emotional arousal	Negative emotional arousal by health consequence	Desire to consume product	Desire to consume product by health consequence	Acceptability	Acceptability by health consequence
Bowel Cancer 1	4.77 (1.47) [1]	4.53 (1.57) *95% CI: 4.42–4.64*	3.08 (2.32) [18]	3.20 (2.37) *95% CI: 3.03–3.36*	3.51 (1.92) [21]	3.65 (1.90) *95% CI: 3.52–3.78*
Bowel Cancer 2	4.65 (1.58) [2]		3.06 (2.27) [19]		3.67 (1.89) [18]	
Bowel Cancer 3	4.18 (1.57) [4]		3.44 (2.48) [3]		3.77 (1.86) [17]	
Breast Cancer 1	4.00 (1.63) [9]	3.86 (1.62) *95% CI: 3.75–3.98*	3.14 (2.28) [17]	3.29 (2.42) *95% CI: 3.12–3.46*	3.89 (1.90) [12]	3.79 (1.88) *95% CI: 3.67–3.93*
Breast Cancer 2	3.93 (1.59) [13]		2.99 (2.16) [21]		3.55 (1.84) [20]	
Breast Cancer 3	3.65 (1.64) [21]		3.73 (2.73) [1]		3.95 (1.89) [8]	
Liver Cirrhosis 1	4.03 (1.76) [8]	4.01 (1.72) *95% CI: 3.89–4.13*	3.23 (2.32) [15]	3.26 (2.33) *95% CI: 3.09–3.42*	3.98 (1.91) [5]	4.01 (1.87) *95% CI: 3.88–4.14*
Liver Cirrhosis 2	3.89 (1.65) [15]		3.23 (2.33) [15]		3.94 (1.87) [9]	
Liver Cirrhosis 3	4.11 (1.74) [6]		3.30 (2.37) [12]		4.10 (1.82) [2]	
Heart Disease 1	4.00 (1.56) [9]	3.97 (1.62) *95% CI: 3.86–4.09*	3.31 (2.31) [11]	3.34 (2.38) *95% CI: 3.18–3.51*	3.91 (1.81) [10]	3.84 (1.80) *95% CI: 3.71–3.96*
Heart Disease 2	4.04 (1.60) [7]		3.44 (2.39) [3]		3.97 (1.76) [6]	
Heart Disease 3	3.88 (1.71) [17]		3.27 (2.43) [13]		3.62 (1.83) *[19)*	
Liver Cancer 1	3.84 (1.59) [19]	4.10 (1.60) *95% CI: 3.98–4.20*	3.63 (2.60) [2]	3.30 (2.43) *95% CI: 3.13–4.37*	3.89 (1.89) [12]	3.95 (1.86) *95% CI: 3.82–4.08*
Liver Cancer 2	4.28 (1.63) [3]		3.00 (2.20) [20]		3.83 (1.91) [16]	
Liver Cancer 3	4.12 (1.54) [5]		3.26 (2.43) [14]		4.12 (1.77) [1]	
Liver Disease 1	3.95 (1.64) [12]	3.91 (1.61) *95% CI: 3.80–4.02*	3.43 (2.49) [5]	3.39 (2.40) *95% CI: 3.22–3.56*	4.05 (1.72) [3]	3.97 (1.78) *95% CI: 3.85–4.10*
Liver Disease 2	3.90 (1.63) [14]		3.36 (2.40) [10]		3.97 (1.83) [6]	
Liver Disease 3	3.89 (1.56) [15]		3.38 (2.32) [8]		3.90 (1.78) [11]	
7 types of Cancer 1	3.83 (1.61) [20]	3.89 (1.60) *95% CI: 3.78–4.00*	3.43 (2.48) [5]	3.41 (2.44) *95% CI: 3.24–3.58*	3.89 (1.75) [12]	3.93 (1.78) *95% CI: 3.81–4.05*
7 types of Cancer 2	3.87 (1.65) [18]		3.42 (2.47) [7]		4.00 (1.73) [4]	
7 types of Cancer 3	3.96 (1.52) [11]		3.37 (2.38) [9]		3.89 (1.86) [12]

**Fig. 3:**
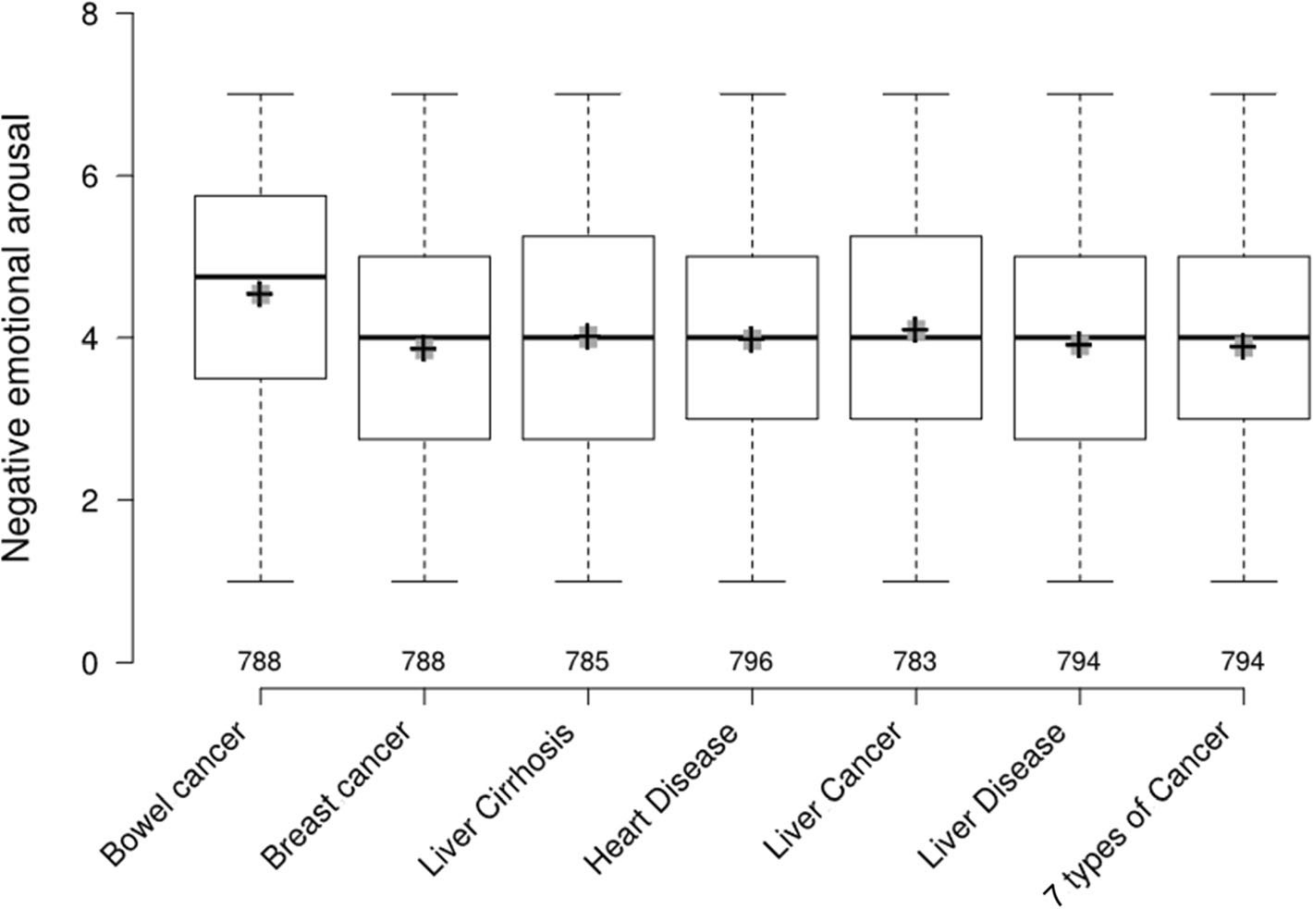
Alcohol: Negative emotional arousal by HWL consequence^1^

#### Food study (see Table 4 and Fig. 4)

The health consequence that elicited the highest NEA was bowel cancer, followed by non-specified cancer. Labels depicting bowel cancer elicited higher levels of NEA than those depicting all other health consequences, with no overlap in 95% CIs. For desire to consume the labelled product, bowel cancer related HWLs were on average rated lowest, though lack of overlap of 95% CIs of their mean values only applied in relation to non-specified cancer and heart disease. HWLs on energy-dense snacks were judged on average more acceptable than on alcohol, with 13 out of 18 snack HWLs rated as at least somewhat acceptable (i.e. with a mean rating above 4). Bowel cancer HWLs were rated on average the least acceptable, with lack of overlap of 95% CIs for mean values indicating lower acceptability than those depicting all other health consequences.

**Table 4:** Food: Means (±SDs) for NEA, desire and acceptability by label and health consequence [ranked from highest to lowest, 1–18]

Label	Negative emotional arousal	Negative emotional arousal by health consequence	Desire to consume product	Desire to consume product by health consequence	Acceptability	Acceptability by health consequence
Bowel Cancer 1	4.59 (1.76) [1]	4.44 (1.76), *95% CI: 4.31–4.56*	3.05 (1.90) [17]	3.08 (1.83) *95% CI: 2.95–3.21*	3.67 (2.04) [18]	3.78 (2.03) *95% CI: 3.64–3.92*
Bowel Cancer 2	4.59 (1.74) [1]		2.91 (1.83) [18]		3.67 (2.05) [18]	
Bowel Cancer 3	4.13 (1.75) [3]		3.27 (1.77) [9]		3.99 (2.00) [14]	
Non-specified Cancer 1	3.93 (1.73) [5]	3.82 (1.74) *95% CI: 3.69–3.94*	3.38 (1.87) [6]	3.39 (1.82) *95% CI: 3.26–3.52*	4.13 (1.89) [11]	4.15 (1.93) *95% CI: 4.02–4.29*
Non-specified Cancer 2	3.82 (1.76) [7]		3.43 (1.86) [4]		4.06 (1.98) [13]	
Non-specified Cancer 3	3.70 (1.73) [12]		3.35 (1.74) [7]		4.27 (1.92) [8]	
13 types of Cancer 1	3.74 (1.77) [11]	3.72 (1.75) *95% CI: 3.60–3.84*	3.23 (1.76) [12]	3.32 (1.83) *95% CI: 3.19–3.45*	3.87 (1.96) [16]	4.12 (1.94) *95% CI: 3.98–4.25*
13 types of Cancer 2	3.60 (1.75) [14]		3.47 (1.86) [2]		4.18 (1.89) [9]	
13 types of Cancer 3	3.82 (1.74) [7]		3.26 (1.86) [10]		4.30 (1.94) [7]	
Type 2 Diabetes 1	3.85 (1.72) [6]	3.65 (1.67) *95% CI: 3.53–3.77*	3.07 (1.71) [16]	3.26 (1.79) *95% CI: 3.13–3.38*	4.42 (1.98) [5]	4.45 (1.93) *95% CI: 4.31–4.58*
Type 2 Diabetes 2	3.78 (1.71) [10]		3.30 (1.88) [8]		4.37 (1.94) [6]	
Type 2 Diabetes 3	3.34 (1.53) [17]		3.40 (1.77) [5]		4.55 (1.89) [1]	
Heart Disease 1	4.03 (1.70) [4]	3.76 (1.78) *95% CI: 3.64–3.89*	3.16 (1.72) [15]	3.35 (1.82) *95% CI: 3.23–3.48*	3.96 (2.03) [15]	4.20 (1.98) *95% CI: 4.06–4.34*
Heart Disease 2	3.66 (1.81) [13]		3.25 (1.84) [11]		4.15 (1.93) [10]	
Heart Disease 3	3.60 (1.81) [14]		3.65 (1.87) [1]		4.48 (1.96) [3]	
Obesity 1	3.12 (1.63) [18]	3.45 (1.79) *95% CI: 3.32–3.57*	3.44 (1.80) [3]	3.29 (1.83) *95% CI: 3.16–3.42*	4.51 (1.89) [2]	4.35 (1.92) *95% CI: 4.21–4.48*
Obesity 2	3.42 (1.77) [16]		3.22 (1.79) [13]		4.43 (1.87) [4]	
Obesity 3	3.81 (1.89) [9]		3.21 (1.90) [14]		4.11 (1.99) [12]

**Fig. 4:**
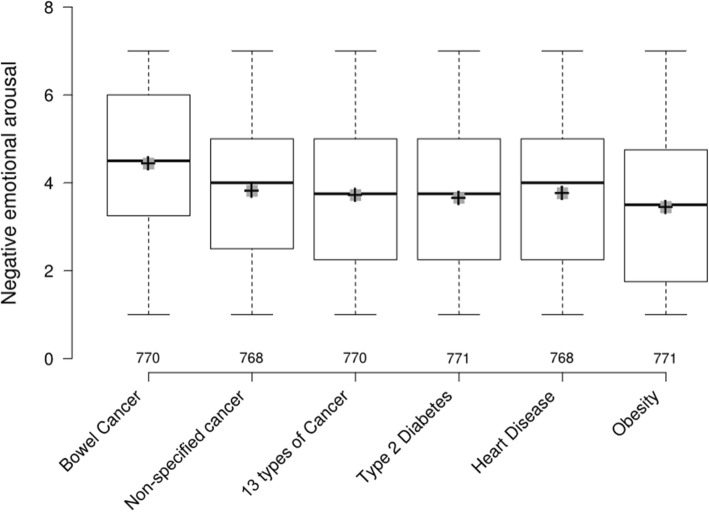
Food: Negative emotional arousal by HWL consequence^.1 1^Centre lines show the medians; box limits indicate the 25th and 75th percentiles; whiskers extend 1.5 times the interquartile range from the 25th and 75th percentiles (no data points exceed this distance, therefore the whiskers are the minimum and maximum values); crosses represent sample means; grey bars indicate 95% confidence intervals of the means. Sample sizes are listed above the x axis

### Content analysis

Three themes were identified in relation to both alcohol and snack HWLs from manual content analysis of participants’ free-text comments:

i. Effectiveness (i.e. whether HWLs were perceived capable of reducing selection and consumption of the products, issues of desensitisation or not attending to HWLs).
ii. Acceptability (i.e. whether HWLs were liked/disliked, supported/opposed, perceived as appropriate or as having any adverse consequences).
iii. Other (including reactions to HWL content, suggestions and comments on general interventions unspecific to HWLs).

HWL-related comments were coded into these themes. Comments could be coded as relating to more than one theme, for example as relating to both acceptability and effectiveness. Additional subthemes were identified, including references to a nanny state, scaremongering, concerns about children’s exposure to HWLs on these products and frustrations regarding mixed heath messages. There was a common sense that HWLs would target the wrong people, spoil treats, and be ignored. Other commonly recurring arguments referred to the right to information, saving the National Health Service and the need for action. Lastly, surprise at the link between alcohol consumption and cancer, especially bowel and breast cancer, was also often expressed.

The analytic procedure as well as a full description of each theme and subtheme, with example comments, is provided in Additional file 1.

### Alcohol study

**Fig. 5:**
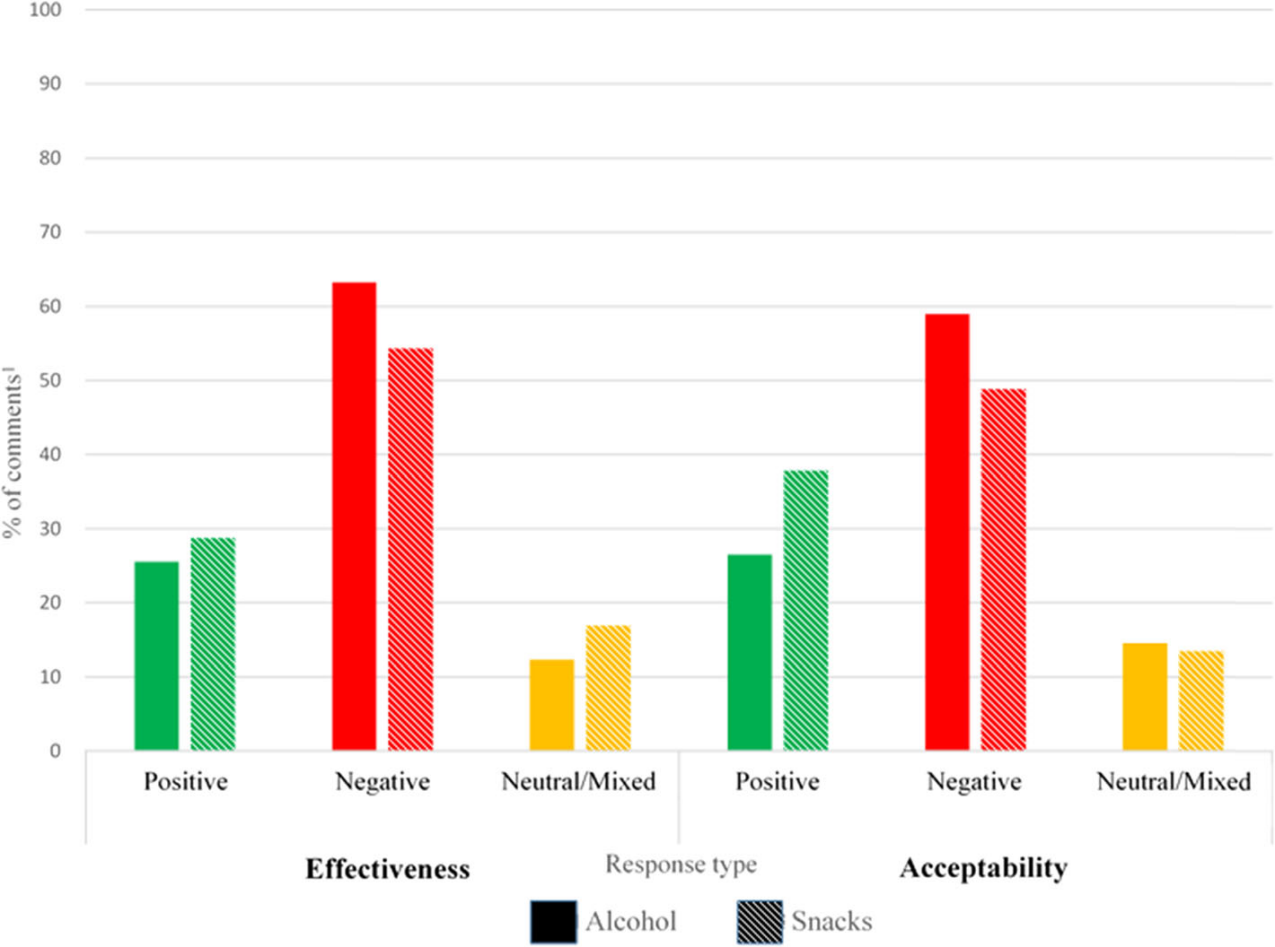
Affective content of comments relating to Effectiveness and Acceptability of image-and-text HWLs on Alcohol and Snack foods. ^1.^ Percentages are of total number of effectiveness and acceptability comments

In total, 460 participants (8.3% of the total sample) made a HWL-related comment. HWL-related responses were coded as positive, negative or neutral/mixed, and into comments related to acceptability or effectiveness. Overall, the majority of comments were negative, with 60.7% coded as negative, and 25.7% positive (see Fig. 5).

#### Effectiveness

Of those participants who commented on effectiveness (*n* = 171), 25.5% felt HWLs would be effective in reducing alcohol consumption.

#### Acceptability

Of those participants leaving an acceptability-related comment (*n* = 234), 26.5% found HWLs on alcohol acceptable.

### Food study

In total, 645 participants (14.0% of the total sample) made a HWL-related comment in the Food study (see Fig. 5). The comments were mixed in sentiment, with a slight majority of negative responses, with 50.9% being coded as negative and 34.3% positive.

#### Effectiveness

Of the participants who commented on effectiveness (*n* = 230), 28.7% felt HWLs would be effective for reducing calorie intake.

#### Acceptability

Of those leaving an acceptability-related comment (*n* = 373), 37.8% found HWLs acceptable on energy-dense snacks.

## Discussion

Image-and-text HWLs portraying bowel cancer elicited the highest levels of negative emotional arousal and lowest desire to consume both alcoholic drinks and energy-dense snacks. They were also the least acceptable of the HWLs investigated. Acceptability was generally low for HWLs applied to alcohol but considerably higher when applied to energy-dense snacks. The majority of free-text comments that were provided – although based on a small subset of participants – were negative for both products.

Given that negative emotional arousal has been identified as a mediator of the effect of HWLs on quit attempts for tobacco [22] and selection behaviour for SSBs [20], the apparent potency of the bowel cancer HWLs in the current study suggests they are the candidate labels with the greatest potential for reducing alcohol and snack food selection and consumption. This is consistent with previous research demonstrating that messages relating to the development of specific cancers are more effective than those referring to non-specified cancer [29], with bowel cancer messages being particularly impactful [36]. Laboratory and field studies using experimental designs are now needed to examine the effectiveness of these HWLs for reducing objectively measured selection and consumption of alcoholic drinks and energy-dense snacks.

In terms of acceptability, HWLs were viewed as less acceptable for alcohol than for snack products. The difference in acceptability may reflect an increased awareness of the adverse effects of excess sugar consumption resulting from an increase in media presence and public discourse of such messages, with recent campaigns raising awareness of the health risks of obesity in adults and children [37] and recent regulatory and legislative activity targeting excess sugar consumption, such as the UK Soft Drinks Industry Levy [38]. By contrast, there has not been an equivalent focus on excess alcohol consumption, with no recent alcohol control policies in England [39]. Furthermore, increased public acceptability for government intervention to protect children [28] may also contribute to HWLs being more acceptable on snack foods.

For both alcohol and snack foods, HWLs depicting bowel cancer were rated the least acceptable. This may be explained by these being the HWLs that also elicited the highest negative emotional arousal and lowest desire to consume the product, and likely related to the anticipated loss of pleasure from such labels suggested in some participants’ open ended responses. The low acceptability of HWLs depicting bowel cancer may also reflect low awareness of the link between this cancer and alcohol and snack foods. Such a lack of awareness has been observed for alcohol and its links with bowel cancer [40] and is consistent with evidence that the alcohol industry downplays the link between alcohol consumption and bowel and breast cancer [32]. By contrast, the HWLs perceived as most acceptable for alcohol and snacks depicted liver cirrhosis and type 2 diabetes, respectively, which may reflect greater public awareness of the association between these conditions and excess consumption of the product [40]. Raising awareness of the links between a range of cancers and excess consumption of alcohol and energy-dense foods through broader health communication approaches could increase awareness and acceptability of related HWLs, and address erroneous perceptions that ‘everything causes cancer’ [41].

Evidence is only one factor that determines the likelihood of policy changes, and so demonstrating effectiveness does not ensure any given intervention will be implemented [25]. A key barrier to implementation is public acceptability [25], which is increasingly recognised for its pivotal role in the extent to which evidence is implemented into policy [26]. As such, were there evidence for the effectiveness of these labels in reducing selection and consumption of alcohol and energy-dense snacks, their low public acceptability may justify generating evidence for other label types. For example, there is indirect evidence suggesting that text-only HWLs can reduce selection of less healthy products - albeit with smaller effect sizes – but are more acceptable [42, 43]. As with the development of HWLs for tobacco, text warnings could potentially be used prior to the introduction of more effective image-and-text HWLs.

### Strengths and limitations

To our knowledge, these are the first robust large-scale studies investigating cognitive and emotional responses to image-and-text HWLs on alcohol products and energy-dense snacks. They provide important initial evidence to inform the development of image-and-text HWLs for reducing consumption of these products, including identifying HWLs that appear most potent for subsequent testing. Participants were from a broad range of ages and SEP, which increases the likelihood that results are generalisable across population groups. Furthermore, while based on comments from only a small subset of participants, the content analysis nonetheless highlighted several issues and reactions relevant to future research or any attempt to implement HWLs.

The current studies have several limitations. While they were designed to assess potential effectiveness by measuring levels of negative emotional arousal, a possible mediator of the impact of HWLs, and desire to consume, providing an indication of urge or intention to consume the product, neither of these measures are able to demonstrate actual effectiveness for changing behaviour. The studies were also conducted online necessitating the use of images of labelled products as opposed to actual products. There is some evidence that participant responses may differ when HWLs are applied to physical products in real-world settings [13, 20]. These studies focused on the perceived effectiveness and acceptability of HWLs across a general population sample. Future research could valuably investigate the impact of labels within different specific population subgroups, for example by age, gender or socio-economic position. Finally, free-text comments were not collected from all participants as they were optional, raising the possibility that the provided comments were unrepresentative.

## Conclusion

The results of these studies identify image-and-text health warning labels that show the greatest potential for reducing consumption of alcoholic drinks and energy-dense snacks. HWLs depicting bowel cancer appear especially promising, although they were the least acceptable. Laboratory and field studies using experimental designs are needed to test their effectiveness for reducing selection and consumption.

## Supplementary information

**Additional file 1. **Content analysis. Subtheme descriptions and examples for the acceptability and effectiveness-related comments in the Alcohol and Food studies.

## Data Availability

The study data can be found on the Open Science Framework: Alcohol study (https://osf.io/pr8zu/); Food Study (https://osf.io/k7tw5/).
