# Supplementary material for: Image-and-text health warning labels on alcohol and food: potential effectiveness and acceptability
Source: BMC Public Health. 2020 Apr 2;20:376. doi: 10.1186/s12889-020-8403-8 (PMC7114781; doi:10.1186/s12889-020-8403-8)
Supplement: Supplementary file 1 — Additional file 1. Content analysis. Subtheme descriptions and examples for the acceptability and effectiveness-related comments in the Alcohol and Food studies. [file 12889_2020_8403_MOESM1_ESM.docx]

| **Supplementary material** |
| --- |

**I. Content analysis**

Analytic procedure

Comments provided by participants were manually coded by two study authors (EP and NC).

*Relevant and irrelevant comments*

Comments were first coded as intervention-related (relevant and included in content analyses) or unrelated (irrelevant and not included in content analyses). Intervention-related comments included those that referred to HWLs, those related to the experience of seeing the HWL during the survey (e.g. change in knowledge, surprise at the content), and suggestions as to how to improve or implement HWLs. They also included more general opinions towards healthcare interventions (e.g. “I support any action against obesity”).

Common examples of irrelevant comments were those relating to general survey experience, general praise which could equally relate to the survey as to the intervention (e.g. “good”), comments on consumption habits and liking or disliking of the chosen product (e.g. “I don’t like that chocolate bar anyway”).

Themes

A list of provisional themes was created by the first author based on an initial viewing of the comments and a preliminary coding scheme was created. Two study authors (EP and NC) coded a random sample of 10% of the comments using this preliminary scheme. The themes were then refined through discussion between the two researchers and a third author (GJH), following which a more detailed coding scheme was created. A further random sample of 10% of the comments was then coded using this refined coding scheme and inter-rater reliability was assessed. This process continued over a further two iterations until the percentage agreement was high (Cohen’s kappa statistic > 0.8). When the final themes and coding scheme had been agreed, the first author coded all of the comments (with interrater reliability of k=.82 (Alcohol) and k=.87 (Food) for the 10% of the comments that were coded by both researchers).

Three main themes were identified: (1) acceptability, (2) effectiveness and (3) other, described below. Additional subthemes were identified (see Results).

*Acceptability*

Acceptability-related comments related to whether the HWLs were considered appropriate and acceptable (irrelevant of whether they would achieve their desired purpose). This included respondents liking or disliking the idea of HWLs, or expressing opposition to or support for introducing HWLs. They also included references to adverse, unintended consequences of introducing HWLs (e.g. how this might affect people with eating disorders, or disturb children), any references to the ‘nanny state’ or impact on personal choice, and references to the believability of the HWL content.

*Effectiveness*

Comments relating to whether the HWLs would work as an intervention (*i.e.* would reduce harmful consumption) were coded as effectiveness-related. This included references to whether people would notice, read or understand HWLs, and whether HWLs would cause people to change their thinking or behaviour in the desired way (e.g. “seeing this would make me think twice” or “I would eat less chocolate if I saw this label”). Effectiveness-related comments also included expressing the risk of desensitisation to HWLs, suggestions that HWLs were “too simplistic” to be a solution, and participants not “seeing the point”, *i.e.* the utility of HWLs.

Comments coded as effectiveness-related, were also divided into subcategories regarding personal effectiveness, i.e. affecting the individual specifically, (e.g. “these labels would definitely put me off eating the chocolate bar”) versus general effectiveness (e.g. “people will just ignore these labels”).

*Other*

HWL-related comments that did not relate to either acceptability or effectiveness were coded as ‘other’. This category was also divided into three subcategories:

1. Reactions to HWL content (e.g. “is it true that breast cancer is caused by alcohol?”)
2. Suggestions (e.g. “I think the warning should be written in red and in bold”’)
3. Comments relating to healthcare interventions in general (e.g. “we need to do more to make the population healthier”).

*Valence of comments*

Each HWL-related comment was further coded as either a: (1) positive, (2) negative or (3) neutral/mixed response to the use of HWLs.

*Double coding of acceptability and effectiveness*

A comment was double coded as effectiveness and acceptability-related, if the themes were equally evoked, and there was no clear dominant theme, or if the valence differed between the themes, e.g. they thought HWLs would work (effective, positive), but at a cost to personal freedom (unacceptable, negative).

Additional details of all themes and subthemes, with example comments, are provided in Table S1.

**Table S1. Subtheme descriptions and examples for the acceptability and effectiveness-related comments in the Alcohol and Food studies**

| **Theme** | **Response type** | **Subtheme** | **Examples comments** | |
| --- | --- | --- | --- | --- |
|  |  |  | **Alcohol Study** | **Food Study** |
| **Acceptability** | **Negative** | **Nanny-state**  References to a ‘nanny-state’ or ‘big brother’ culture, and a feeling of being patronised and manipulated was common. These responses were most often the most hostile and angry comments. References to the nanny state were more common in the Alcohol study. | *“Do not even consider putting these types of labels on alcoholic beverage bottles. This is a ridiculous level of nanny state behaviour and complete lack of making people take responsibility for their own actions.”*  *“If you encourage companies to put disgusting pictures on my alcohol I will rip the labels off in the supermarket and deposit them at customer services. I will take my beer home without obnoxious pictures on […] leave us alone”*  *“What next? “Don’t breath its bad for you", "don't live its bad for you", "don't enjoy your life its bad for you", f**k off Nanny State...”* | *“Stop trying to police our eating habits. […] We don't need to live in a nanny state […]I personally would eat extra chocolate and snacks if these images were introduced on labels just to prove a point that I won't be controlled by people who think they can tell others how to live their lives!”*  *“[…] an invasion and violation of personal liberties. If someone stood physically next to me with this kind of image while I was enjoying some chocolate I might well punch them in the face. I might also call the police and tell them the person with the image was causing a breach of the peace”* |
|  |  | **Children’s exposure**  There was a recurring theme that HWL were inappropriate, and potentially traumatising, for children, especially in the Food study where the target product is something children buy. On the other hand, a few participants suggested this intervention should be introduced specifically for children. | *“I would not like to see these graphic pictures of effects of drinking alcohol on bottle labels. Written warnings are acceptable, however when most adults with families take their young children shopping with them I do not feel it is appropriate for them to be exposed to the photographs.”* | *“I think it's disgusting that you would choose to subject children to these images which is unacceptable and could cause them distress.”*  *“I would be concerned about children seeing these sort of pictures on snacks - especially young ones.”*  *“I don't fully oppose the use of these labels and warnings however I think there may be some ethical issues with it. For example, the graphic content so readily available to children from a young age?”* |
|  |  | **Graphic image is too much**  Strongly linked to the concern of children’s exposure was the pictorial element of the HWL, which was considered too graphic or unnecessary. In both the Alcohol and Food study, it was commonly suggested that text-only warnings would be more acceptable. | *“I am not sure that manufacturers would support the graphic labelling depicted. Perhaps textual details of typical symptoms of disease associated with drinking might be more acceptable”*  *“I think a written warning on an alcoholic beverage is fine, an image of the possible damage of harm that might be done is totally unnecessary”*  *“A written warning maybe, but do not want to see that picture on a bottle of wine that may sit on my dinner table.”* | *“A text only warning would be more suitable as the child can then have a discussion about what the warnings mean, but putting images like that on chocolate bars and the like is totally irresponsible.”*    *“It would be fine to put a warning label but the picture is a bit far to put on a bar of chocolate.”* |
|  |  | **Scaremongering**  There was a common perception of HWLs being unacceptable due to spreading fear unnecessarily, being  overly negative, over-the-top and  even cruel. Arguments based on  comparisons with cigarettes were  very common, most likely because  the HWLs resembled those used on  cigarette packs. A popular argument  was that snack HWLs were  unacceptable as food consumption is incomparable to smoking. | *“Drinking alcohol does not CAUSE cancer. This is pure scaremongering”*  *“Scaremongering is what I call this. If I want to drink I will”*  *“I think the labels are too much and unfair on those who drink sensibly having to look at them”* | *“There are surely better, more positive, ways to encourage moderation without resorting to fear and guilt mongering. Unlike cigarettes, high calorie foods/snacks are not inherently dangerous and can be useful and a bit of psychological relief for people who are on a low income or otherwise have a tight budget. Putting distressing images on an affordable luxury seems rather cruel.”* |
|  |  | **Spoiling treats**  A common theme was that HWLs would ruin any pleasure consuming these products, which often are regarded as well-deserved treats. The risk of de-valuation or ‘cheapening’ of the appearance of these products, often associated with gifts, was also presented as an issue. | *“Wine can be quite valuable and is used as a gift as well. It would be embarrassing to go to a dinner party with a great big cancer sticker on the bottle!”*  *“It’s almost spoiling that little bit of a pleasure once in a while […] Making life more depressing with all the austerity already present in this country so please don't take this one step further and cause more depression”*  *“If those off putting label are added to bottles of alcohol you will punish and disgust normal, decent occasional drinkers and kill the pleasure of consuming responsibly and in a cultured way.* | *“If you are giving someone a box of chocolates for their birthday you would not want that on there and people are entitled to a treat without being made to feel bad.”*  *“It seems a shame to spoil the enjoyment of something that can be enjoyed as an occasional treat”*  *“I think the labelling spoils the idea of chocolate being a treat and would have to be carried across all chocolate products, which would ruin a lovely big box of chocolates for Christmas.”* |
|  |  | **Believability**  Believability came into question regularly, especially with the cancer-related HWLs. Several participants also suggested that everything caused cancer, highlighting the danger of the warned outcome becoming perceived as seemingly inevitable and therefore not worth trying to avoid. | *“Everything seems to cause cancer and illness these days so it just gets a bit tedious”*  *“According to one study or another, everything causes cancer. So, if I enjoy eating or drinking something I'm not going to stop consuming it because of a warning label on it.”* | *“I support putting warnings on such products but that was over the top maybe a smaller label with warnings about calories and risk of diabetes or heart disease but warning about cancer from eating a chocolate bar is wrong!”* |
|  |  | **Confusion**  Comments complaining of the  confusion caused by mixed health and social messages were common. They depicted a confusing picture, which might ultimately allow consumers to pick which arguments they would rather adhere to. | *“everybody knows that smoking means inhaling tar which is carcinogenic, but the general message about alcohol is unfocused and often contradictory (e.g. all alcohol consumption is bad for you because alcohol is a poison - yes- but also moderate consumption of red wine can be beneficial) and it is patronising/misleading to ignore one message in favour of another”* | *“It would be better to address the mixed messages that are being sent regarding health (should we be 'body-positive' or cut calories?)”* |
|  | **Positive** | **Need for action**  These positive comments were mostly themed around a need for action that was currently lacking, and suggestions that policy makers were currently too afraid or simply incapable of tackling the obesity crisis effectively. This was often backed up by arguments that if HWLs were on cigarettes, they should be on other health-harming products too. | *“I do think there should definitely be warnings on alcohol bottles/cans etc. They have them on cigarettes so I think this is no different and may discourage people from drinking too much.”* | *“I think is / was the best / most hard hitting I've ever seen! I love this! I've struggled for many, many years with obesity [...] I think it's about time someone had the balls to do this. Well done! I for one salute you! Too much nampy pamby around obesity...”* |
|  |  | **Right to information**  A recurring theme in positive acceptability was people’s rights to have this warning information, in order to make informed choices. These participants did not view HWLs as imposing rules, but simply providing information. | *“I think anything that can have an adverse effect on your health should have a clear warning on it to give people an informed choice as there is so much marketing to persuade young people that it is safe with no risk”* | *“It is just providing advice not saying do not eat it, that is up to the person. Do not see a problem with it”* |
|  |  | **Saving the NHS**  Saving costs and pressure on the NHS was a very common  argument in favour of the  intervention in the Food study.  This argument did not  come up in the Alcohol study. | N/A | *“I support the labelling in an effort to reduce the cost to the country of obesity and Type 2 diabetes”*  *“I think it's great that this type of deterrent is being looked at particularly knowing how much money obesity costs the NHS and the fact it is also a high cause of cancer.”* |
| **Effectiveness** | **Not effective** | **Wrong target**  There was a common feeling that the HWLs would not affect those most in need of changing their consumption. These comments were often  presented alongside a sense of  injustice at being unfairly punished  due to other people’s lack of willpower. | *“I feel like putting labels on alcohol products won't stop addicts from using these products, it will punish responsible drinkers who may only drink socially on the weekend”* | *“This sort of label will only be read and acted on by people who are already aware of the dangers. It is aimed at the wrong people.”* |
|  |  | **Desensitisation**  Another common subtheme was the issue of desensitisation, with arguments often based around comparisons with participants’ experience of cigarette pack HWLs. | *“Changing cigarette packs didn't bother people, once you've seen an image it loses its shock value, people joke about them rather than take them seriously.”* | *“Whilst obesity is becoming more and more common, I'm not sure putting this kind of imagery on food packaging will make that much of a difference. Whilst the images are graphic and quite unpleasant I feel like people can easily get used to, and desensitised to imagery like this. As much as the imagery is unappealing, if I wanted a bar of chocolate, this would not put me off eating it.”* |
|  |  | **Old information**  Another recurring subtheme was that the warning information is already well known, and that as we already know the information, seeing it on the packet will not make any difference. | *“I think alcohol warning labels, like tobacco warning labels are really quite silly/futile. I can't imagine many people don't know that there can be adverse health effects from drinking alcohol and I don't suppose that people that want to drink (again, like smoking) will be put off by a warning label. I certainly wouldn't be. I'd just try to collect all the different labels.”* | *“Like with cigarettes/tobacco, I personally don't think warnings/pictures will put people off consuming the product. Everyone KNOWS sweets/chocolate/crisps are bad for you but we still eat them. I can't see a picture making any difference.”* |
|  | **Effective** | **Deterrent at point-of-purchase**  Other comments suggested that while they did already know the information, seeing the labels at the point of purchase could be an extra deterrent – highlighting the distinction between HWLs providing information, but also working as a nudge. These comments were often supported by arguments based on comparisons with cigarette HWLs. | *“I think it's a brilliant idea to add those kind of warning labels to alcohol bottles […] I reckon it'd be more effective that the labels on cigarettes as smoking is an addiction whereas most people drinking alcohol aren't addicted, meaning that they're more likely to read the label and feel more empowered to choose not to purchase any.”* | *“I enjoy snacks and realise the health implications as I am a Nurse. Even so I think the labels would help me reduce my intake of unhealthy snacks.”*  *“I think it’s a brilliant idea […] I would say having this on them would definitely be a bit more of a deterrence and give me more will power - especially because they are usually an impulse buy would make you think twice about what you’re putting in your body”* |
|  |  | **Deterrent at point-of-consumption**  Other positive comments suggested that seeing the HWLs would put them off and make them less likely to consume the product. | *“I quite like the idea of putting health warnings on alcohol the same as cigarettes, I think it would be more impactful[.] with cigarettes you are not constantly looking at the packet but with a bottle of beer it is always in eye view”* | *“I think it is very logical to put this on packets and may make people think twice about consuming the snack; it certainly made me think twice”* |
|  |  | **Surprise at cancer-link**  Contrary to the recurring argument for perceived ineffectiveness of HWLs (i.e. that people already know the warning information), positive responses suggested some of the information (specifically the cancer-based information) was new, suggesting providing this information may be worthwhile. This was most common in the Alcohol study. | *“I didn't ever think alcohol could be directly linked with cancer, like I think cigarettes are. […] including images like this on bottles of wine for example would really make me think twice about drinking!”*  *“was unaware of a link between drinking and breast cancer”*  *“Having the picture of bowel cancer on a bottle of wine is very off putting - having wine with a meal is usually seen as a treat but finding out it can cause cancer puts a new light on it. So many people have been touched by cancer in many ways - I have a feeling that if this label was displayed on alcohol, sales might plummet, as many people are trying to eat healthily today and no-one wants cancer. Certainly think this association between alcohol and bowel cancer should be more widely publicised...”* | *“The association with cancer to obesity is new to me and does cause concern/may change my eating habits.”* |
